# Comparative Genome Analysis and Phenotypic Characterization of *Clostridium gasigenes* CGAS001 Isolated From Chilled Vacuum-Packed Lamb Meat

**DOI:** 10.3389/fmicb.2020.02048

**Published:** 2020-08-24

**Authors:** Joseph Wambui, Nicole Cernela, Sabrina Corti, Roger Stephan

**Affiliations:** Institute for Food Safety and Hygiene, Vetsuisse Faculty, University of Zurich, Zurich, Switzerland

**Keywords:** *Clostridium gasigenes*, blown pack spoilage, chilled meat, genome analysis, antibiotic resistance, toxins, polyketides

## Abstract

Genomic data for psychrophilic bacteria causing blown pack spoilage (BPS) are limited. This study characterizes the genome of a novel *Clostridium gasigenes* strain CGAS001 isolated from meat juice sample (MJS) of vacuum-packed lamb meat by comparing it with the type strain *C. gasigenes* DSM 12272 and five strains representing four other BPS-causing *Clostridium sensu stricto* species. Phenotypic characteristics of the strain, which include biochemical characteristics, antimicrobial resistance and production of putative polyketide, have been determined. The size of its draft genome is 4.1 Mb with 3,845 coding sequences, 28.7% GC content and 95 RNA genes that include 75 tRNAs, 17 rRNAs, and 3 ncRNAs. Average Nucleotide Identity (ANI) and digital DNA–DNA Hybridization (dDDH) predict that *C. gasigenes* CGAS001 and DSM 12272 constitute a single species (ANI and dDDH = 98.3% for speciation) but two distinct subspecies (dDDH = 73.3% for subspeciation). The genome is characterized by saccharolytic, lipolytic and proteolytic genes as well as hemolysins and phospholipases, which are consistent with its phenotype. The genome also reveals the ability of *C. gasigenes* to synthesize polyketides which is demonstrated by the antimicrobial activity of a crude polyketide extract against *Listeria monocytogenes* and *Enterococcus devriesei*. The strain is resistant to polymyxin B and streptomycin. The genetic and phenotypic analyses suggest that CGAS001 constitutes a novel subspecies of *C. gasigenes* adapted to a saprophytic lifestyle and can synthesize narrow spectrum antimicrobial compounds.

## Introduction

*Clostridium gasigenes* is a psychrophilic anaerobic Gram-stain-positive bacterium and a member of *Clostridium sensu stricto*, which causes a type of meat spoilage commonly referred to as blown pack spoilage (BPS) (Broda et al., 2000). The spoilage is characterized by gas production and pack distention usually occurring without pack integrity or temperature abuse (Wambui and Stephan, 2019). Since the first confirmed case of BPS caused by *C. gasigenes* in New Zealand (Broda et al., 2000), the species has been detected and/or isolated from BPS in other countries including Ireland (Moschonas et al., 2009; Bolton et al., 2015) and Brazil (Silva et al., 2011). *C. gasigenes* is, together with *Clostridium estertheticum*, one of the main causes for BPS in vacuum packed and refrigerated meat (Reid et al., 2017; Dorn-In et al., 2018). Other causative agents include *Clostridium algidicarnis*, *Clostridium frigidicarnis*, and *Clostridium tagluense* (Adam et al., 2010; Cavill et al., 2011).

As a strict anaerobe, *C. gasigenes* would not persist on meat surfaces in aerobic conditions in the vegetative state, hence it is likely its spores are the major contaminants of meat within the slaughterhouses. Within these premises, the main reservoirs for *C. gasigenes* have been suggested to include soil, hides and feces (Broda et al., 2002, 2009). Upon contamination, vacuum packing and cold storage then provide conditions that are necessary for spore germination and finally BPS (Wambui and Stephan, 2019). Ten spores of *C. gasigenes* seem to be enough to cause BPS in vacuum packed meat (Moschonas et al., 2010), with the BPS occurring within 36 days or 56 days at 1°C depending on applied meat processing techniques (Moschonas et al., 2011b). The spores are therefore a primary target for reducing the occurrence of BPS (Moschonas and Bolton, 2013). Data have shown, that oxygen exposure is a possible option to prevent growth of germinated *C. gasigenes* spores (Adam et al., 2013). Besides this, no other data are available in regards to *C. gasigenes* spore characteristics, including resistance mechanisms or germinants (Adam et al., 2010).

The exact mechanisms by which *C. gasigenes* and the other psychrophilic anaerobic *Clostridium* spp. cause BPS remain largely unknown (Húngaro et al., 2016). However, it has been suggested that lactate utilization is important for BPS inception especially by *C. estertheticum* (Yang et al., 2010). Lactate is also a germinant for *C. estertheticum* spores (Rajagopal et al., 2016). On the other hand, *C. gasigenes* does not utilize lactate (Yang and Badoni, 2013), instead, lactate is one of its metabolites (Broda et al., 2000). Therefore, there exist metabolic processes and possibly spore germination mechanisms that are fundamentally different between *C. gasigenes* and *C. estertheticum* through which they cause BPS. Further studies on how psychrophilic clostridia cause BPS, including the identification of functional genes, have been suggested (Adam et al., 2010; Yang et al., 2014).

Up to now, BPS data is limited to phenotypic studies. In the current genomic era, it is imperative that phenotypic BPS studies are supplemented by genomic-based studies. Universal systems for gene knockout, including ClosTron (Heap et al., 2007) and RiboCas (Cañadas et al., 2019) that allow effective exploitation of Clostridial genome data have already been developed. Utilization of these systems can unravel the missing information regarding metabolic processes, spore characteristics and other features that are vital for BPS prevention. Other unknown characteristics including toxins and antimicrobial compounds such as those encoded in the genome of *C. estertheticum* (Yu et al., 2016) can be identified and studied. Beyond BPS and the public safety concerns, genome data can unravel new class of compounds including antimicrobials that have therapeutic use. Presently, only a few of these compounds have been reported for *Clostridium* spp. (Pahalagedara et al., 2020). There is therefore a need for amassing and mining genome data for the unexploited psychrophilic *Clostridium* species.

In this study, the whole genome sequence of *C. gasigenes* strain CGAS001, isolated from vacuum packed lamb meat, was characterized. Including the genomes of the type strains *C. gasigenes* DSM 12272 (Broda et al., 2000) and five selected strains representing four other BPS-causing *Clostridium sensu stricto* species, provided an excellent opportunity for the first comprehensive comparative analysis involving psychrophilic anaerobic *Clostridium* spp. causing BPS. Valuable insights into the lifestyle, metabolic diversity, public health significance and secondary metabolites of the strain have been uncovered.

## Materials and Methods

### Bacterial Isolation and Culturing

Ten meat juice samples (MJS) from a recent study (Wambui et al., 2020) were investigated. The MJS were positive for anaerobic psychrophilic *Clostridium* spp. after an initial real-time PCR (RT-PCR) screening. Isolation was carried out anaerobically in a multi-step approach involving spores recovery and elimination of competitive microflora by heat or ethanol treatment followed by enrichment and plating as previously described (Moschonas et al., 2009). Briefly, 1 ml of each positive MJS was mixed with 9 ml pre-reduced peptone-yeast-glucose-starch (PYGS) medium (Broda et al., 2000) and divided into two. One set was heated at 80°C for 10 min while the other set was mixed with 50% ethanol (v/v) for 1 h at 4°C. Both sets were incubated anaerobically for 3 weeks at 4°C after which, they were serially diluted and plated on Columbia Agar supplemented with 5% defibrinated sheep blood (CBA). The plates were incubated anaerobically for 3 weeks at 4°C. Colonies with previously described characteristics (Moschonas et al., 2009) were selected and purified for 2 weeks at 4°C. Representative colonies were gram stained and screened by RT-PCR using the primers for psychrophilic *Clostridium* spp. (Brightwell and Clemens, 2012). One isolate from ethanol treated regime, whose cells appeared as long thick rods with or without spores and stained blue and genomic DNA was positive for psychrophilic *Clostridium* spp., was selected for whole genome sequencing.

### Genome Sequencing, Analysis, Assembly and Annotation

DNA was isolated from a culture grown anaerobically for 10 days at 4°C on CBA using the DNeasy Blood and Tissue Kit (Qiagen, Hilden, Germany) and eluted in 10 mM Tris pH 7.3. The DNA concentration was measured with a Nanodrop (Witec AG, Switzerland). Sequencing libraries were prepared using the Illumina Nextera DNA Flex and sequenced on an Illumina MiniSeq (Illumina, San Diego, CA, United States) with a minimal coverage of 50×. The length of the library was 150–300 bp. After quality control with FastQC^1^, the reads were assembled with SPAdes v. 3.12.0. The quality of the genome was further determined *in silico* using CheckM v.1.018 (Parks et al., 2015) and Contest16S (Lee et al., 2017). The genome was annotated online in RAST^2^ (Brettin et al., 2015) and NCBI Prokaryotic Genome Annotation Pipeline (Tatusova et al., 2016).

### Strain Identification

The identification of the strain was initially performed by the 16S rRNA gene using the EzBioCloud^3^ web server (Yoon et al., 2017a). Phylogenetic analyses were further carried out by comparing the 16S rRNA gene of the isolate with the 16S rRNA gene sequences of other *Clostridium sensu stricto* spp. strains known to cause BPS namely; *C. gasigenes* DSM 12272, *C. algidicarnis* NCFB 2931, *C. bowmanii* DSM 14206, *C. estertheticum* DSM 8809, *C. frigidicarnis* SPL77A, *C. frigoris* DSM 14204, *C. frigorophilum* 14F, and *C. tagluense* A121. The 16S rRNA sequences were aligned using the progressive alignment algorithm and a phylogenetic tree created from the aligned sequences in CLC Workbench Genomics v. 8.1 (Qiagen, Aarhus, Denmark) using the neighborhood joining tree construction method while applying the Jukes-Cantor nucleotide substitution model. Bootstraps were based on 1,000 replicates with other parameters set to default. The sequences were downloaded from the National Center for Biotechnology Information (NCBI) database^4^ (NCBI Resource Coordinators, 2018).

The delimitation of the isolate by its whole genome sequence (WGS) was carried out *in silico* through digital DNA–DNA hybridization (dDDH) using DMSZ’s Genome-to-Genome Distance Calculator^5^ web server (Meier-Kolthoff et al., 2013) against WGS of *C. gasigenes* DSM 12272 (Genbank GCA_900104115.1) and five strains from four BPS-causing clostridia *sensu stricto*. Three species were represented by one strain each namely, *C. algidicarnis* B3 (Genbank; GCA_000703125.1), *C. frigidicarnis* DSM 12271 (Genbank; GCA_900111985.1) and *C. tagluense* A121 (Genbank; GCA_003865095.1) while *C. estertheticum* was represented by two strains; *C. estertheticum* subsp. *estertheticum* DSM 8809 (Genbank; GCA_001877035.1) and *C. estertheticum* subsp. *laramiense* DSM 14864 (Genbank; GCA_008933175.1). The WGS of the strains were downloaded from the NCBI database (NCBI Resource Coordinators, 2018). Furthermore, the Average Nucleotide Identity (ANI) of the isolate’s WGS was compared against the WGS of the same clostridia strains using EzBioCloud^6^ web server (Yoon et al., 2017b). The genome of the isolate was also compared to and visualized against the genomes of *C. gasigenes* DSM 12272, *C. algidicarnis* B3, *C. frigidicarnis* DSM 12271, and *C. estertheticum* subsp. *estertheticum* DSM 8809 using BLAST Ring Image Generator (BRIG) software v. 0.95 (Alikhan et al., 2011).

### Gene Identification

Virulence factors of the isolate were identified using VFDB 2019^7^ web server (Liu et al., 2019). PlasmidFinder v. 2.1^8^ and ResFinder v. 2.1^9^ (Zankari et al., 2012) servers was used to identify genes linked to plasmids and antimicrobial resistance, respectively. The pathogenicity potential of the strain in humans was determined using PathogenFinder v. 1.1^10^ web server (Cosentino et al., 2013). Blast search analyses were performed for unique gene sequences using NCBI Blast server^11^ while the domains and topography of unique proteins encoded by the genes were predicted using NCBI Conserved Domain Database^12^ (Marchler-Bauer et al., 2009) and Phobius^13^ web servers, respectively. Detailed identification of metabolic genetic elements, other genes/proteins of interest and the comparison of these components with BPS causing *Clostridium* spp. strains was carried out in RAST (Brettin et al., 2015).

### Genome Comparison and Interpretation of Data From RAST

A one versus all genomes comparison strategy was carried out in RAST (Brettin et al., 2015). The full list of annotated coding sequences (CDS) using RAST for both *C. gasigenes* strains CGAS001 (strain identified in this study) and DSM 12,272 (type strain) were summarized in two Excel files. First, only the sequences of both CGAS001 and DSM 12272 (Supplementary Tables S1, S2, respectively) were compared and presented with either strain serving as the reference strain and the other as the test strain. The output data included columns for contig number (Contig), gene number (Gene), gene identity (Gene ID), amino acid length of encoded protein (Length) and gene function (Function) for both strains. Additionally, a column for a homologous gene’s percent identity (Percent ID) to a reference strain’s gene was included. Lastly, CGAS001, as a reference strain, was compared with the five strains from four *Clostridium* spp. causing BPS mentioned above (Supplementary Table S3). The output data comprised of contig number (Contig), gene number (Gene), gene identity (Gene ID), amino acid length of encoded protein (Length) and gene function (Function) only for the reference strain. Only percent identity (Percent ID) of homologous genes to the reference strain’s genes was included for the test strains. Reverse comparison for each of the *Clostridium* spp. test strain with CGAS001 was carried out individually (data no shown). In all outputs, CDS locus was based on the RAST annotation and are continuously referenced in this manuscript using the following example: CGAS001 peg.1 and DSM 12272 peg.10 referring to CDS loci 1 and 10 in strains CGAS001 and DSM 12272, respectively.

### Biochemical Characterization of *Clostridium gasigenes* CGAS001

For biochemical characterization, cultures of CGAS001, grown on CBA for 72 h at 22°C, were standardized to 3.0 MacFarland, inoculated in respective media and incubated anaerobically at 22°C for 5–7 days. Saccharolytic and proteolytic assays were carried out on PYGS agar (PYGS medium with 1.5% agar) and Nutrient agar with 2% Skim milk, respectively. Lipolytic and lecithinase activities were determined on PYGS agar supplemented with 1% egg-yolk emulsion. For further biochemical profile characterization and acidification potential, API20A kit (bioMeriéux, Marcy l’Etoile, France) was used following manufacturer’s specifications. All reagents in this section and subsequent sections were purchased from Sigma-Aldrich Chemie GmbH, Buchs, Switzerland, unless stated otherwise. All experiments were carried out in three biological replicates.

### Antibiotic Resistance Profile of *Clostridium gasigenes* CGAS001

For antimicrobial resistance testing, an inoculum prepared as described above was spread with a sterile swab stick on Brucella Blood Agar. The testing was carried out by disk diffusion method against eight antibiotics; tetracycline (30 μg), chloramphenicol (30 μg), polymyxin B (300 μg), streptomycin (10 μg), clindamycin (2 μg), erythromycin (15 μg), penicillin (10 U), and vancomycin (30 μg). The antibiotic disks were purchased from Becton Dickinson AG, Allschwil, Switzerland. Zones of inhibition were measured to nearest mm. The antibiotic resistance tests were carried as per EUCAST guidelines for hemolytic bacteria and interpreted using the EUCAST guidelines for when standards are unavailable for a species^14^, which is the case for *C. gasigenes*. All experiments were carried out in three biological replicates.

### Screening of *Clostridium gasigenes* CGAS001 for Antimicrobial Activity

The antimicrobial potential of CGAS001 was determined using the agar streak method (Almalki, 2020) with modifications. Briefly, a fresh culture of CGAS001 prepared as described above was streaked on CBA plates and incubated anaerobically for 72 h at 22°C. Strains to be tested, *L. monocytogenes* EGDe (Glaser et al., 2001) and *Escherichia coli* grown aerobically on Brain Heart Infusion (BHI) agar and Luria-Bertani (LB) agar, respectively, for 24 h at 37°C were standardized to 0.5 McFarland and streaked perpendicular to CGAS001 on the CBA plates. The plates were incubated aerobically at 37°C for 24 h. Antimicrobial activity was determined as lack of growth by *L. monocytogenes* and *E. coli* near the streaked line of CGAS001. Where growth was absent, the zone of inhibition was measured to the nearest mm. All experiments were carried out in three biological replicates.

### Extraction and Characterization of Putative Polyketide From *Clostridium gasigenes* CGAS001

#### Extraction of Putative Polyketide

The extraction of crude polyketide was carried out by the solid agar method (Arasu et al., 2008) with modifications. Briefly, cultures grown as described above were standardized to 0.5 MacFarland, spread on the entire surface of 10 CBA agar plates using sterile swabs and incubated anaerobically at 22°C for 15 days. After incubation, the agar (together with the grown culture) was cut into small cubes and pooled into a 1,000 ml beaker into which 150 ml acetonitrile was added. The agar and the culture were left to soak for an hour then centrifuged at 12,000 × *g* for 30 min. The supernatant was collected, and the residue resuspended in 75 ml acetonitrile then treated as before. The resulting supernatants were pooled together, and the volume increased to 400 ml with acetonitrile. This resulted in formation of precipitates, which were separated by centrifuging two times (8,000 × *g*; 5 min). The crude extract was produced from the supernatant using a rotary evaporator at 30°C and stored in parafilm-sealed 5 ml Eppendorf tube at 4°C for future use.

#### Antimicrobial Activity of Putative Polyketide

The antimicrobial activity of the crude polyketide extract was determined by disk diffusion method (Voitsekhovskaia et al., 2020) with modifications. Briefly, the crude extract was suspended in distilled water (0.5 mg/μl) and 50 μl of the mixture loaded on sterile 6 mm disks (Whatman^®^, United States). The loading was done in two subsequent steps of 25 μl and drying under a hood for 2 h. *L. monocytogenes* EGDe and *E. coli* were grown as described above. *E. devriesei* K8-ED (Wambui et al., 2018) and *Staphylococcus aureus* RN4220 (Kreiswirth et al., 1983) were grown aerobically on BHI agar and Trypticase Soy agar, respectively, for 24 h at 37°C. The two strains were standardized to 0.5 McFarland and spread on respective media and the dried disks placed on top. The plates were incubated at 37°C and antimicrobial activity determined as presence of clear zones around the disks. Where inhibition zones were present, they were measured to the nearest mm. All experiments were carried out in three biological replicates.

#### Physical Characteristics of Putative Polyketide

The physical activity of the crude polyketide extract was determined as previously described (Kuiper et al., 2004) with modifications. For the effect on the crude extract on surface tension of water, the crude extract was suspended in distilled water (20 μg/ml) and divided into two. Into one portion, crystal violet dye was added for visual contrast. To ensure that the dye had no effect on the surface tension, distilled water dyed with crystal violet was used as a control. Samples, 500 μl, were pipetted into 1 ml cuvettes and the cuvettes left to stand in atmospheric air for 30 min at room temperature. Further effects of the extract on surface tension were carried out on non-polar surface using the drop collapse assay. 25 μl of dyed distilled water with (20 μg/ml) or without the crude extract was dropped on the surface of parafilm and left to stand at room temperature for 30 min. The effect of the crude extract on water’s meniscus in the cuvettes and collapse of the drop on parafilm was determined visually. For hygroscopic assay, 30 μg of the crude extract was placed on a microscope slide cover glass and left to stand in atmospheric air for 12 h at room temperature. The hygroscopic potential was determined as the percentage change of the extract’s weight after 12 h. All experiments were carried out in three independent replicates.

### Data Analysis

Data for biochemical characteristics of CGAS001 and physical characteristics of its putative polyketide extract were described qualitatively. Zones of inhibitions for both antibiotic resistance profile of CGAS001 and the antimicrobial sensitivity of indicator strains to putative polyketide as well as the changes in weight of the putative polyketide after exposure to air were described as means and standard deviation. Where applicable, means were compared using the *t*-test (*p* = 0.05).

## Results

### Isolation of an Anaerobic Psychrophilic *Clostridium* spp. Strain

Culturing of anaerobic psychrophilic *Clostridium* spp. was successful in one out of the 10 meat juice samples (MJS). The positive sample (ID = 1399/12) was MJS from vacuum packed lamb meat imported to Switzerland from New Zealand. Purified isolates from the sample formed gray, semi-translucent, raised, convex, shiny smooth circular colonies with entire margins that were β-hemolytic on Columbia blood agar supplemented with 5% sheep blood (CBA). Colonies were visible on CBA after 7 days of incubation in anaerobic conditions at 4°C. Under the microscope (100×), the isolates formed thick rods that stained blue. Subterminal spores were also visible in a few cells. On CBA, the isolates failed to grow at 25, 30, and 37°C in both aerobic and anaerobic conditions and at 4°C in aerobic conditions. A single typical colony from the ethanol treated sample was chosen for genome sequencing.

### Features of the Draft Genome

The draft genome sequence of the isolate was assembled into 56 contigs and its size and Glycine-Cysteine (GC) content estimated to be 4.1 Mb and 28.7%, respectively. CheckM (Parks et al., 2015) predicted low levels of contamination in the genome (0.81%). No foreign 16S rRNA fragments were detected by Contest16S (Lee et al., 2017). The annotated genome had 3,845 coding sequences. Ninety-five RNAs that included 9, 4 and 4 5S, 16S and 23S rRNAs, respectively, 75 tRNAs and 3 ncRNAs were predicted. The sequences of the four partial 16S rRNA genes were identical. No plasmids related genes were predicted.

### Genotypic Identification of Novel *Clostridium gasigenes* Strain CGAS001

**FIGURE 1:**
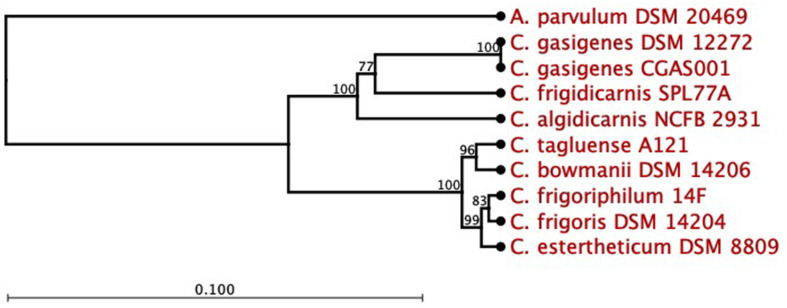
Phylogenetic tree of *Clostridium gasigenes* CGAS001 by 16S rRNA gene sequence. *C. gasigenes* CGAS001 and DSM 12272 formed a phylogenetic subunit distinct from selected blown pack spoilage causing *Clostridium sensu stricto* spp. strains. *Atopobium parvulum* DSM 20469 was used as the outgroup. Number at joint indicated bootstrap values (%) while the bar indicates 0.1 substitutions per nucleotide position. Phylogenetic tree reconstructed by CLC Workbench Genomics based on alignment of the nucleotide sequences.

**FIGURE 2:**
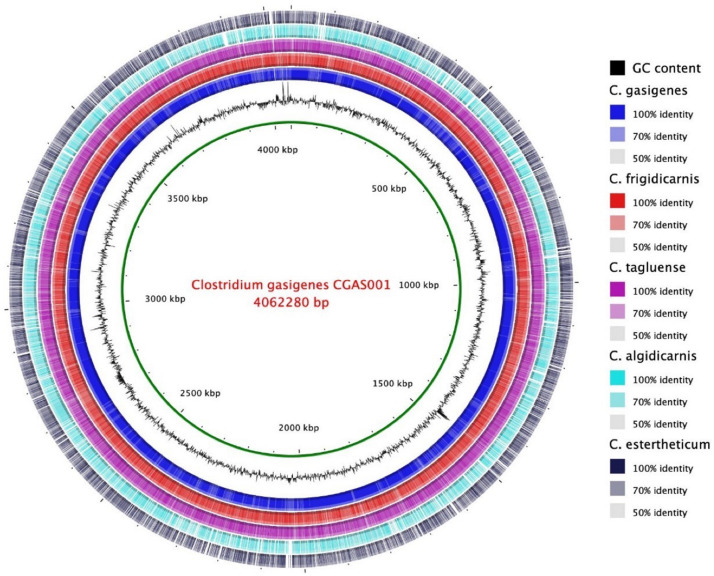
Circular genomic map of *C. gasigenes* CGAS001 and selected genomes of five *Clostridium* species causing blown pack spoilage. There was a closer identity of CGAS001 strain CGAS001 to the only other sequenced *C. gasigenes* strain DSM 12272 than the other selected *Clostridium* species. The circular map produced using BRIG software.

The 16S rRNA partial sequence of isolate, 1,319 bp (88.9% complete), was 100% identical to that of *C. gasigenes* type strain DSM 12272 at a coverage of 83%. A 16S rRNA-based phylogenetic analysis of the two strains and seven BPS causing *C. sensu stricto* spp. revealed the isolate and *C. gasigenes* DSM 12272 clustered together (Figure 1). The two strains formed a larger mono-phylogenetic unit with *C. algidicarnis* NCFB 2931 and *C. frigidicarnis* SPL77A. At the whole genome sequence (WGS) level, the Average Nucleotide Identity (ANI) values between the isolate and six *Clostridium* spp. revealed the relationship, at 98.3% was closest between the isolate and *C. gasigenes* DSM 12272 while in the other tested *Clostridium* spp., the relationship ranged between 76.1 and 70.7% (Table 1). Moreover, the digital DNA–DNA hybridization (dDDH) value between the isolate and the DSM 12272 was 98.3%, which was above the 70% threshold for a single species. On the other hand, the probability that the isolate and *C. gasigenes* DSM 12272 were related to the same subspecies was 73.3%, which is below the 79% threshold for single subspecies. The relation of the genomes of the isolate to *C. gasigenes* DSM 12272 and selected WGS of psychrophilic *Clostridium* spp. causing BPS is presented in Figure 2. Based on these genotypic considerations, the isolate from sample 1399/12 was identified as a member of *C. gasigenes* species and assigned the strain name CGAS001. The isolate is henceforth referred by the name *C. gasigenes* CGAS001.

**TABLE 1:** The average nucleotide identity (ANI) and digital DNA–DNA hybridization (dDDH) values calculated from the genomes of *Clostridium gasigenes* CGAS001 against five other strains from “Blown pack” spoilage causing *Clostridium* spp.

Strains^†^	ANI value (%)*	dDDH value (%)^§^	
		For same species	For same subspecies
*C. gasigenes* DSM 12272	98.3	98.3	73.3
*C. frigidicarnis* DSM 12271	71.8	0	0
*C. tagluense* A121	71.4	0	0
*C. estertheticum* DSM 14864	71.2	0	0
*C. estertheticum* DSM 8809	70.8	0	0
*C. algidicarnis* B3	70.7	0	0

^†^C. gasigenes CGAS001 served as the reference strain. *Calculated using OrthoANIu algorithm in EzBioCloud web server. The ANI cut-off value for the same species is ≥ 95% (Yoon et al., 2017b). ^§^Calculated using logistic regression (Formula 2) using Genome-to-Genome Distance Calculator web server. The subspecies dDDH cut-off value for the same species and subspecies are ≥70% and ≥79–80%, respectively (Meier-Kolthoff et al., 2013).

### General Metabolic Features

#### Saccharolytic Features

Both *C. gasigenes* CGAS001 and DSM 12272 encoded one α-amalyse (CGAS001 peg.659), one pullulanase (CGAS001 peg.977) and three neopullulanases (CGAS001 peg.980, 1572, 3743, and 3749) (Supplementary Table S1). *C. gasigenes* CGAS001 and DSM 12272 had 17 and 19 genes, respectively, encoding phosphotransferase system (PTS) component IIC of the PTS systems. All of these could be categorized into six of the seven major PTS subclasses namely; glucose, fructose, lactose, galactitol, mannose, and L-ascorbate families. CGAS001 encoded one xylanase (CGAS001 peg.512) (Supplementary Table S1) unlike DSM 12272, which did not (Supplementary Table S2), but paled in comparison to *C. estertheticum* representative strains that had 7 to 9 putative xylanases. Saccharolytic activity of CGAS001 was demonstrated by a positive starch hydrolysis test. Furthermore, the API20A kit demonstrated the strain’s ability to utilize and produce acids in different carbohydrates among them D-mannitol, D-maltose, D-salicin, D-xylanose, D-cellobiose, D-mannose, D-melezitose, and D-trehalose. Both CGAS001 and DSM 12272 encoded five chitinases among which, four (CGAS001 peg.620-623) formed a cluster that is present in *C. gasigenes* species (Supplementary Table S1), and absent in genomes of the other clostridia strains.

#### Lipolytic, Lecithinase and Proteolytic Features

CGAS001 encoded four phospholipases (CGAS001 peg.290, 308, 1564, and 1959) (Supplementary Table S1) compared to three phospholipases in the DSM 12272 (Supplementary Table S2). In the other *Clostridium* spp., only *C. frigidicarnis* DSM 12271 (n = 5) encoded more phospholipases. There were five lipases/esterases in strain CGAS001 (Supplementary Table S1) compared to four in strain DSM 12272 (Supplementary Table S2). Consistent with the genome, CGAS001 showed both lipolytic and lecithinase activities. Genes coding for proteolytic enzymes were present in the genome of strain CGAS001, including a microbial collagenase (CGAS001 peg.634) and Alpha-clostripain precursor (CGAS001 peg.1562) (Supplementary Table S1). Preliminary phenotypic characterization showed CGAS001 had proteolytic activity on skim milk. A putative cysteine protease (CGAS001 peg.3272: herein referred to CysP for Cysteine Protease) (Supplementary Table S1), which was predicted to have a signal peptide, was identified. *cysP* gene was present and localized adjacent to *recX* gene in *C. gasigenes* 12272 (Supplementary Table S2) and the other *Clostridium* spp. genomes. CysP protein sequences in all five *Clostridium* species shared a conserved GEW motif in the C-terminal.

### Virulence Factors

**FIGURE 3:**
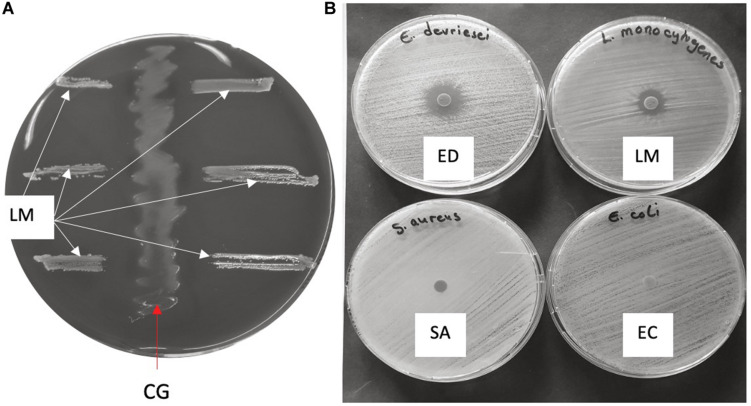
Antimicrobial activity of *C. gasigenes* CGAS001 crude polyketide extract. **(A)** The potential of CGAS001 to synthesize antimicrobial compounds was initially identified using the agar streak method whereby the growth of *Listeria monocytogenes* EGDe (LM) was inhibited by CGAS001 (CG). **(B)** The crude extract showed narrow spectrum antimicrobial activity against selected Gram-positive and negative bacteria using the disk diffusion method. The extract was active against *L. monocytogenes* EGDe and *Enterococcus devriesei* K8-ED (ED), but inactive against *Staphylococcus aureu*s (SA) and *Escherichia coli* (EC).

Twenty-two potential virulence factors grouped into 16 classes were predicted in *C. gasigenes* CGAS001 (Table 2). A putative clostridiolysin biosynthetic gene cluster (CGAS001 peg.1962-1970) was identified in CGAS001 (Supplementary Table S1). The same gene cluster was present in DSM 12272 (Supplementary Table S2). The presumptive clostridiolysin-like protein precursor (CGAS001 peg.1962) was 51 aa long in both strains, but CGAS001 had an extra 50 aa encoded protein (CGAS001 peg.1961) adjacent to the precursor (Supplementary Table S1). Among the other *Clostridium* spp. strains, the clostridiolysin-like biosynthetic cluster was only identified in *C. algidicarnis* B3 and *C. frigidicarnis* DSM 12271 with the latter encoding two more proteins (52 and 53 aa) adjacent to its precursor (52 aa) (Figure 3). A CDS for an exfoliative toxin A (CGAS001 peg.1361) was also identified in the genomes of both *C. gasigenes* strains (Supplementary Tables S1, S2) as well as, *C. frigidicarnis* DSM 12271 and *C. tagluense* A121. Despite of these virulence factors, strain CGAS001 was determined to be a non-human pathogen (probability = 0.2). Similar predictions were made for *C. gasigenes* DSM 12272 strain whose probability was 0.3.

**TABLE 2:** Potential virulence factors in the genome of *Clostridium gasigenes* CGAS001*.

VF class	Virulence factors	Related genes
Adherence	Fibronectin-binding protein	*Fbp*
	GroEL	*gro*EL
	LPS O-antigen (*Pseudomonas aeruginosa*)	*wbp*I
Regulation	CheA/CheY (*Listeria*)	*che*Y
	LisR/LisK (*Listeria*)	*lis*R
	VirR/VirS (*Listeria*)	*vir*R
Toxin	Alpha-clostripain	*clo*SI
	Hemolysin	Undetermined
		Undetermined
Copper uptake	Copper exporter (*Mycobacterium*)	*ctp*V
Glycosylation system	O-linked flagellar glycosylation (*Campylobacter*)	*neu*B2
Immune evasion	Capsule (*Staphylococcus*)	*cap*F
		*cap*G
		4I
	Polysaccharide capsule (*Bacillus*)	*gal*E
		*gta*B
Intracellular survival	Sugar-uptake system (*Listeria*)	*hpt*
Iron uptake	Periplasmic binding protein-dependent ABC transport systems (*Vibrio*)	*vct*C
Lipid and fatty acid metabolism	Pantothenate synthesis (*Mycobacterium*)	*pan*D
Magnesium uptake	Mg^2+^ transport (*Salmonella*)	*mgt*B
Motility	Flagella (*Helicobacter*)	*fli*P
Others	O-antigen (*Yersinia*)	*ddh*A
Phagosome arresting	Nucleoside diphosphate kinase (*Mycobacterium*)	*ndk*
Secretion system	Type III secretion system (*Chlamydia*)	*cds*N
Serum resistance and immune evasion	LPS (*Francisella*)	*wbt*E
Stress adaptation	Catalase-peroxidase (*Mycobacterium*)	*kat*G

*Identified in Virulence Finder Database (VFDB) 2019 web server.

### Antibiotic and Stress Resistance Genes

Annotation in RAST web server identified 53 genes coding for stress resistance in *C. gasigenes* CGAS001. Some of the roles of protein encoded by these genes are listed in Table 3. These included resistance to tetracycline, chloramphenicol, beta lactamases and fluoroquinolone. There were also proteins involved in arsenic, cadmium, cobalt-zinc-cadmium and copper resistance. No known chloramphenicol resistance proteins were annotated in DSM 12272 (Supplementary Table S2). A protein annotated as “Daunorubicin resistance transmembrane protein” (CGAS001 peg.632) was also identified (Supplementary Table S1). Domain search revealed the protein was an ABC-2 type transporter (*E*-value = 2.19e^–37^), hence forming an ABC transporter gene cluster with two other proteins annotated as “Efflux ABC transporter, permease protein” (CGAS001 peg.631) and “Efflux ABC transporter, ATP-binding protein” (CGAS001 peg.633) in a what seems to be a gene cluster of 3 ABC transporters. Further analysis revealed this cluster was only present in *C. gasigenes* DSM 12272 (Supplementary Table S2) and *C. algidicarnis* B3. Although antibiotic resistance genes were encoded in the genome of CGAS001, the strain showed high sensitivity to all antibiotics tested (zones of inhibition >30 mm) apart from polymyxin B and streptomycin, which had no effect on the strain’s growth.

**TABLE 3:** Stress resistance subsystems in the genome of *Clostridium gasigenes* CGAS001*.

Subsystem	Role
Arsenic resistance	Arsenical pump-driving ATPase (EC 3.6.3.16)
	Arsenical resistance operon *trans*-acting repressor ArsD
Beta-lactamase	Beta-lactamase repressor BlaI
	Beta-lactamase (EC 3.5.2.6)
Cadmium resistance	Cadmium-transporting ATPase (EC 3.6.3.3)
	Cadmium efflux system accessory protein
Cobalt-zinc-cadmium resistance	DNA-binding heavy metal response regulator
	Cobalt-zinc-cadmium resistance protein
	Transcriptional regulator, MerR family
Copper homeostasis: copper tolerance	Copper chaperone
	Copper-translocating P-type ATPase (EC 3.6.3.4)
	Multicopper oxidase
	Cytoplasmic copper homeostasis protein CutC
Multidrug Resistance Efflux Pumps	Macrolide export ATP-binding/permease protein MacB (EC 3.6.3.-)
	Multidrug-efflux transporter, major facilitator superfamily (MFS) (TC 2.A.1)
	Multi antimicrobial extrusion protein [Na (+)/drug antiporter], MATE family of MDR efflux pumps
Resistance to fluoroquinolones	Efflux pump Lde
	DNA gyrase subunit A (EC 5.99.1.3)
	DNA gyrase subunit B (EC 5.99.1.3)
Resistance to Vancomycin	Vancomycin B-type resistance protein VanW
Streptothricin resistance	Streptothricin acetyltransferase, Streptomyces lavendulae type
Tetracycline resistance, ribosome protection type	Ribosome protection-type tetracycline resistance related proteins, group 2
	Translation elongation factor G
Zinc resistance	Response regulator of zinc sigma-54-dependent two-component system

*Annotated in RAST web server.

### Spore Related Coding Sequences

Proteins related to spore stress resistance, namely six small acid soluble spore proteins (SASPs), were identified (Supplementary Table S1). Three of the SASPs (61 aa each) were in a gene cluster (CGAS001 peg.1080–1082). Fourth, fifth and sixth SASPs were 61, 60, and 84 aa long (CGAS001 peg.1166, 1736 3249, respectively). The sequences of the six CGAS001 SASPs were 100% identical to those of *C. gasigenes* 12272 (Supplementary Table S1). Only *C. estertheticum* strains encoded equal or more (*n* = 6 or 7) SASPs than the two *C. gasigenes* strains. Furthermore, gene clusters for at least three SASPs were identified in *C. estertheticum* representative genomes whereby four SAPS clustered together. Both strains CGAS001 and *C. gasigenes* 12272 possessed similar sporulating proteins, most of which were 100% conserved (Supplementary Table S1). The only distinction between the two strains was a presumptive *cotJ* operon (CGAS001 peg.3684–3686) coding for proteins annotated as “Protein CotJA,” “Polypeptide composition of the spore coat protein CotJB,” and “Manganese catalase (EC 1.11.1.6) ≥ Spore coat protein CotJC.” The presumptive operon was only present in *C. gasigenes* CGAS001. The other *Clostridium* spp. strains had the *cotJABC* genes clusters apart from *C. frigidicarnis* DSM 12271. In *C. estertheticum*, two separate clusters of *cotJ* operons were found.

### Cryptic Polyketide Synthesis Genes

**FIGURE 4:**
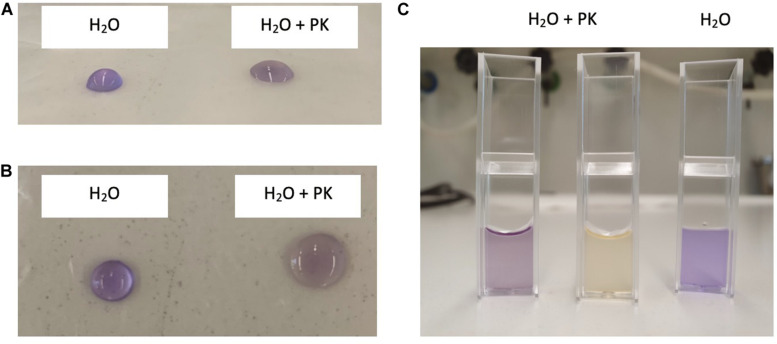
Biosurfactant activity of *C. gasigenes* CGAS001’s crude polyketide extract. Addition of the crude extract to distilled water (20 μg/ml) affected the surface tension of the water. Distilled water without the extract was used as a control. Samples were left to stand in atmospheric air for 30 min at room temperature. Crystal violet was added to the mixture for visual effect and had no influence on the surface tension of the distilled water. **(A)** Front view and **(B)** top view of 25 μl droplets of distilled water with (H_2_O + PK) or without (H_2_O) the extract on a non-polar surface (parafilm) after 30 min. **(C)** Five hundred microliter of distilled water with (H_2_O + PK) or without (H_2_O + PK) the polyketide extract in 1 ml cuvettes after 30 min.

*Clostridium gasigenes* CGAS001 harbored genes involved in the biosynthesize of polyketides (Supplementary File S1). The genes were localized in a cluster of more than 60 encoded proteins some of which, including nine proteins polyketide synthase modules and related proteins, a two-component system and seven ABC type transporters, are listed in Table 4. Similar clusters with proteins annotated as polyketide synthase modules and related proteins, were only identified in *C. frigidicarnis* DSM 12271, which encoded twenty proteins. The presence of a bioactive putative polyketide was initially identified phenotypically by inhibitory activity against *L. monocytogenes* EGDe through the cross-streak assay (zone of inhibition = 10.6 ± 0.5 mm) (Figure 3A). Furthermore, the crude extract showed antimicrobial activity against *L. monocytogenes* EGDe and *E. devriesei* K8-ED (zones of inhibition = 12.6 ± 0.5 and 16.0 ± 0.0 mm, respectively) (Figure 3B), with *E. devriesei* being more inhibited than *L. monocytogenes* EGDe (*p* < 0.05). The crude extract showed no activity against *S. aureus* RN4220 and *E. coli* (Figure 3B). Besides the antimicrobial activity, the putative polyketide demonstrated biosurfactant activity as determined by its effect on water surface tension evident in the collapse of water droplets (Figures 4A,B) and effect on the meniscus of distilled water with the compound (Figure 4C). Furthermore, the extract was hygroscopic following 3.5 ± 0.5% increase in weight after 12 h exposure to air (*p* < 0.05).

**TABLE 4:** Proteins, among them polyketide synthesis proteins, encoded by a gene cluster within the genome of *Clostridium gasigenes* CGAS001*.

Contig	Gene	Length (aa)	Predicted protein
22	3765	220	Macrolide export ATP-binding/permease protein MacB
23	3766	1554	Polyketide synthase modules and related proteins
23	3767	637	ABC transporter, permease/ATP-binding protein
23	3768	252	ABC transporter-like sensor ATP-binding protein
23	3769	229	Putative thioesterase
23	3770	560	Efflux ABC transporter, permease/ATP-binding protein
24	3774	323	sensor histidine kinase
24	3775	175	ABC transporter-like sensor linked response regulator
24	3776	39	hypothetical protein
24	3777	238	ABC transporter, ATP-binding protein
24	3778	829	ABC transporter, permease/ATP-binding protein
24	3779	339	Polyketide synthase modules and related proteins
25	3780	3025	Polyketide synthase modules and related proteins
33	3814	1130	Polyketide synthase modules and related proteins
35	3818	898	Polyketide synthase modules and related proteins
36	3819	710	Polyketide synthase modules and related proteins
36	3820	171	Long-chain-fatty-acid–CoA ligase
37	3821	156	Long-chain-fatty-acid–CoA ligase
37	3822	610	Polyketide synthase modules and related proteins
38	3823	339	Mobile element protein
38	3824	168	hypothetical protein
39	3825	574	Polyketide synthase modules and related proteins
40	3826	442	Mobile element protein
41	3827	517	Polyketide synthase modules and related proteins
42	3828	311	Mobile element protein
43	3829	251	hypothetical protein
43	3830	169	Long-chain-fatty-acid–CoA ligase

*Annotated in RAST web server.

## Discussion

*Clostridium gasigenes* is among the important and recognized causative agents for BPS of refrigerated vacuum-packed meat (Broda et al., 2000; Moschonas et al., 2011a; Silva et al., 2011). Genomic studies for the species are nonetheless lacking. In this regard, the present study characterized the genome of a novel *C. gasigenes* strain CGAS001 isolated from meat juice sample of vacuum-packed lamb meat imported to Switzerland from New Zealand by comparing it with the type strain *C. gasigenes* 12272 and five other clostridia strains from four BPS-causing *Clostridium sensu stricto* species namely; *C. algidicarnis* B3, *C. estertheticum* subsp. *estertheticu*m DSM 8809, *C. estertheticum* subsp. *laramiense* DSM 14864, *C. frigidicarnis* DSM 12271 and *C. tagluense* A121. The successful isolation of the *C. gasigenes* CGAS001 occurred in one of the 10 real time-PCR positive samples identified in a recent study (Wambui et al., 2020).

*In silico* methods namely, average nucleotide identity (ANI) and digital DNA–DNA Hybridization (dDDH) have been widely applied to identify bacteria whereby for a bacteria species, the cut-off values for ANI and dDDH are ≥95% and ≥70%, respectively (Meier-Kolthoff et al., 2013, 2014; Chun et al., 2018). The ANI values between *C. gasigenes* CGAS001 and *C. gasigenes* DSM 12272 were above the threshold for a species, thus the two could be affiliated to the same species. On the other hand, the subspecies dDDH cut-off value for a similar bacteria subspecies is ≥79–80% (Meier-Kolthoff et al., 2014). The value for the strains CGAS001 and DSM 12272 was below the subspecies dDDH threshold (Table 1), indicating two subspecies delineation for both strains. Hence CGAS001 can be regarded as a novel subspecies of *C. gasigenes*.

*Clostridium gasigenes* occurs in numerous environments (Moschonas et al., 2009; Silva et al., 2011) requiring a multitude of metabolic enzymes for successful habitat colonization. Direct or indirect contact of meat with these environments may influence contamination of meat with the bacteria. In this regard, a combined action of several saccharolytic enzymes is necessary for complete degradation of starch (Sebaihia et al., 2007), hence the identification of α-amalyse, pullulanase and neopullulanases suggest that strain CGAS001 is cable of complete starch hydrolysis and can inhabit starch rich environment. This was validated by the starch hydrolysis assay showing that CGAS001 could hydrolyze starch similar to DSM 12272 (Broda et al., 2000). In same previous study, strain DSM 12272 did not utilize xylose (Broda et al., 2000), which was confirmed by the absence of xylanase in its genome (Supplementary Table S2). Conversely, xylose was utilized by CGAS001 using the API20A test kit, which can be attributed to the presence of a xylanase in its genome. Xylanases hydrolyze xylan, a hemicellulose that is highly abundant in nature (Walia et al., 2017). The ability of CGAS001 to utilize other saccharides may result from the PTS systems encoded by its genome. A key feature of the CGAS001, which was also reported for other *C. gasigenes* strains (Silva et al., 2011), was its inability to utilize glucose.

Lipolytic and proteolytic enzymes have a strong effect on the quality of meat and other proteinaceous and fatty foods in general. Enzymes secreted by psychrotrophic and psychrophilic bacteria can cause spoilage at refrigeration temperatures (von Neubeck et al., 2015; Zhu et al., 2017), thus the identification of lipases encoded by CGAS001 and the confirmation of their activities support the existence of complex enzymatic systems in CGAS001 necessary for proliferation in meat and potential to cause meat spoilage. On the other hand, a putative cysteine protease, CysP, with a signal peptide in the N-terminus suggests the ability of CGAS001 to express proteases into the extracellular milieu, which was demonstrated by proteolytic activity on casein in the Nutrient agar supplemented with skim milk. Furthermore, a conserved WEG motif in CysP’s C-terminus in four *Clostridium* spp. is suggestive of a functional importance of the motif, but so far, the protein has not been characterized.

Several toxins, among them hemolysins/phospholipases and clostidiolysin-like encoding genes affirm both the hemolytic and lecithinase phenotypes of CGAS001 identified in the type strain DSM 12272 (Broda et al., 2000). The presence of the hemolysins and lecithinases was consistent with hemolysis on CBA and lecithinase activity on PYGS agar supplemented with egg yolk emulsion. On the other hand, a CDS annotated as Phospholipase/lecithinase/hemolysin that was unique only to the genomes of CGAS001 (Supplementary Table S1) and it had a homologous coding gene *C. tagluense* A121. It is unknown whether encoded proteins confer unique phenotypes in these strains. Despite of this, pathogenic potential predictions made presently for both *C. gasigenes* strains and the earlier *in vivo* assay for DSM 12272 (Broda et al., 2000) suggest that *C. gasigenes* species is non-pathogenic to humans.

CGAS001 possess several antimicrobial resistance genes (ARGs) (Table 3), similar to the report for *C. estertheticum* DSM 8809 (Yu et al., 2016). The presence of resistance genes does not always correspond to phenotypic responses (Eitel et al., 2013). This was confirmed by the antimicrobial resistance assay whereby CGAS001 was phenotypically only resistant against streptomycin and polymyxin B. As previously reported, polymyxin B has no activity against Gram-positive and anaerobic bacteria (Zavascki et al., 2007). However, this is the first report of streptomycin resistance in a BPS-causing clostridia species. The resistance mechanisms and the risk of transfer of the resistance to other bacteria warrant further investigation. Non-pathogenic organisms have been reported to transfer ARGs to pathogenic bacteria of clinical significance (Rossolini et al., 2008).

Small acid soluble proteins (SASPs) confer moist heat, UV radiation, hydrogen peroxide, hydrochloric acid, nitrous acid and formaldehyde resistance to clostridial spores (Raju et al., 2007; Paredes-Sabja et al., 2008). Their identification in the genome of CGAS001 and other BPS causing *Clostridium* spp. makes the SASPs potential biomarkers that can be targeted as a means of controlling BPS. Spore coats, including CotJABC proteins, are also important for spore germination and/resistance (Henriques et al., 1995). Previously, only *Bacillus cereus* and *Bacillus subtilis* were reported to encode all CotJABC proteins (Henriques and Moran, 2007; Aronson, 2018), despite a previous study showing an ORF adjacent to *cotJB* was co-transcribed with *cotJB* and *cotJC* in *Clostridium novyi*-NT (Bettegowda et al., 2006). The present study led to the identification of putative CotJA encoded in the genome of CGAS001 (Supplementary Table S1) and other *Clostridium* spp. strains indicating that the strains possess all CotJABC proteins thus affirming the study in *C. novyi*-NT. The fact that *C. gasigenes* DSM 12272 (Supplementary Table S2) and *C. frigidicarnis* DSM 12271 lack the *cotJ* operon indicted its presence is variable among *Clostridium* spp.

The genome of CGAS001 revealed cryptic gene clusters for polyketide biosynthesis. Polyketides are natural products widely used for their antibiotic, anticancer, immunosuppressive, and cholesterol-lowering properties (Shimizu et al., 2017). Presently, we have shown that CGAS001 produces a putative polyketide, which is consistent with its genome analysis. Production of secondary metabolites is an adaptation by producer strains to effectively compete in respective niches. The demonstration that CGAS001 encodes and produces polyketide with antimicrobial activity, although at a narrow spectrum, shows one of the adaptive features that may enhance its ability to grow and cause spoilage in meat. Further characterization indicated the polyketide has both hygroscopic and biosurfactant properties. The identification of orphan putative polyketide synthase modules in *C. frigidicarnis* DSM 12271 further makes the BPS-causing clostridia a promising niche for the discovery of novel metabolites, including antimicrobial compounds. In *Clostridium* spp., only a limited number of antimicrobial products have been identified, isolated and characterized (Pahalagedara et al., 2020). Further research involving purification and detailed characterization of the polyketide will be carried out in future by our laboratory.

## Conclusion

Whole genome sequence analysis determined that *C. gasigenes* CGAS001 and DSM 12272 constitute a single species, but are genetically distinct at subspecies level, making this the first report for a possible subspeciation in *C. gasigenes*. Metabolically, CGAS001 is saccharolytic, lipolytic and proteolytic. A xylanase encoding gene and ability to utilize xylose distinguish CGAS001 from the type strain DSM 12272. Genes for tetracycline, chloramphenicol, beta lactamases and fluoroquinolone resistance are present, but no phenotypic resistance was observed for the antibiotics. However, the strain is resistant to both polymyxin B and streptomycin. The genome is further characterized by toxin encoding genes among them phospholipases/haemolysins/lecithinases whose activity has been observed *in vitro*. CGAS001 has potential for novel antimicrobial synthesis owing to the nine identified orphan polyketide synthase modules and the confirmation of antimicrobial activity of a crude polyketide extract against *L. monocytogenes* and *E. devriesei*. Future molecular and phenotypic studies will be undertaken to characterize the functional roles for the genes identified.

## Data Availability Statement

The whole-genome shotgun project of the CGAS001 strain has been deposited in GenBank under the accession number JAAVWW010000000. The version described in this paper is the first version, accession number JAAVWW010000000.

## Author Contributions

RS, JW, and SC designed the study. RS supervised the study. JW and NC performed the experiments. JW performed genomic analyses. JW and RS wrote and revised the manuscript. All authors contributed to the article and approved the submitted version.

## Conflict of Interest

The authors declare that the research was conducted in the absence of any commercial or financial relationships that could be construed as a potential conflict of interest.

## Footnotes

1 http://www.bioinformatics.babraham.ac.uk/projects/fastqc/

2 https://rast.nmpdr.org/rast.cgi

3 https://www.ezbiocloud.net/

4 https://www.ncbi.nlm.nih.gov/

5 https://ggdc.dsmz.de/ggdc.php#

6 http://www.ezbiocloud.net/tools/ani

7 http://www.mgc.ac.cn/cgi-bin/VFs/v5/main.cgi?func=VFanalyzer

8 https://cge.cbs.dtu.dk/services/PlasmidFinder/

9 https://cgE.cbs.dtu.dk/services/ResFinder/

10 https://cge.cbs.dtu.dk/services/PathogenFinder/

11 https://blast.ncbi.nlm.nih.gov/Blast.cgi

12 https://www.ncbi.nlm.nih.gov/Structure/cdd/wrpsb.cgi

13 http://phobius.sbc.su.se/

14 https://www.eucast.org/ast_of_bacteria/guidance_documents/
